# A Pilot Study Evaluation of 3-Dimensional Imaging in Cosmetic Breast Augmentation: Results of a Single Surgeon 3.5-Year Retrospective Study Using the BREAST-Q Questionnaire

**DOI:** 10.1093/asjof/ojab005

**Published:** 2021-01-25

**Authors:** Lauren E Hutchinson, Andrea D Castaldo, Cedar H Malone, Nicole Z Sommer, Ashley N Amalfi

**Affiliations:** Southern Illinois University School of Medicine, Springfield, IL, USA; Southern Illinois University School of Medicine, Springfield, IL, USA; Southern Illinois University School of Medicine, Springfield, IL, USA; Southern Illinois University School of Medicine, Springfield, IL, USA; Southern Illinois University School of Medicine, Springfield, IL, USA

## Abstract

**Background:**

Traditional methods of breast implant size selection provide limited ability to demonstrate postoperative outcomes. Three-dimensional (3D) imaging provides an opportunity for improved patient evaluation, surgical planning, and evaluation of postoperative breast appearance.

**Objectives:**

The authors hypothesized that preoperative 3D imaging for patients undergoing breast augmentation would improve patient satisfaction and understanding of expected surgical outcomes.

**Methods:**

A retrospective review of patients undergoing breast augmentation by a single surgeon over a 3.5-year period was performed. Patients presenting after the VECTRA was purchased had preoperative 3D imaging, while patients presenting before this did not. Eligible patients received a BREAST-Q questionnaire designed for postoperative evaluation of breast augmentation. They also received a second survey that evaluated expected vs actual breast outcomes.

**Results:**

In total, 120 surveys were mailed and 61 patients (50.8%) returned the survey. The 3D imaged group had improved BREAST-Q scores regarding satisfaction with outcome, surgeon, and physical well-being compared with the group that did not. The imaged group also had higher size, shape, and overall breast correlation scores, confidence in implant size selection scores, and communication with surgeon scores. The differences between the 2 groups were not statistically significant.

**Conclusions:**

Three-dimensional imaging is a valuable tool in breast surgery. Although this study showed improvement in patient satisfaction and predicted outcome scores in the 3D imaged group, the results were not statistically significant. With the majority of patients reporting that they would choose 3D imaging, it appears to instill confidence in patients regarding both surgeon and implant selection.

**Level of Evidence: 4:**

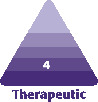

Breast augmentation is the most common cosmetic surgical procedure performed in the United States with over 299,715 performed in 2019.^1^ During the preoperative consultation for breast augmentation, the patient is expected to make a variety of decisions regarding implant type, implant size, pocket position, and incision location. The surgeon guides the decision-making process and the extent of patient involvement in the final decision is highly variable.

Three-dimensional (3D) imaging is a relatively new and promising technology in the field of plastic surgery. This technology may be used in a variety of procedures, including breast surgery, facial plastic surgery, and body contouring. Studies have shown that 3D imaging techniques can accurately assess breast volume preoperatively. Three-dimensional imaging can also quantitatively measure breast asymmetry in terms of volume, shape, and location of the inframammary fold.^2-5^ This allows for asymmetries identified with 3D imaging to be highlighted during the surgical consultation.

Patients value the consultation process as a time to establish rapport with their surgeon and delineate their expectations regarding surgery.^6^ The goal of the preoperative consultation is shared decision making, which involves awareness of choices, understanding of the pros and cons of a surgery, and determination of postoperative goals.^7^ Three-dimensional imaging provides patients an opportunity to take a more active role in decision making for breast augmentation surgery. The purpose of this pilot study was to determine if preoperative 3D imaging improves the breast augmentation consultation. We hypothesized that preoperative 3D imaging would improve patient satisfaction and understanding of expected surgical outcomes.

## METHODS

### Augmentation Consultation

At our institution, an in-person consultation is first performed in the examination room in which goals regarding size, shape, and pocket placement are discussed. The patient also “tries on” different implant sizes by placing them in her unpadded bra to determine an estimated implant size. She is then taken for 3D imaging where she views projected outcomes with a variety of implant sizes. The appointment concludes once the patient and surgeon are in agreement on implant characteristics that best achieve the patient’s goals.

### Three-Dimensional Imaging System

**Figure 1.**
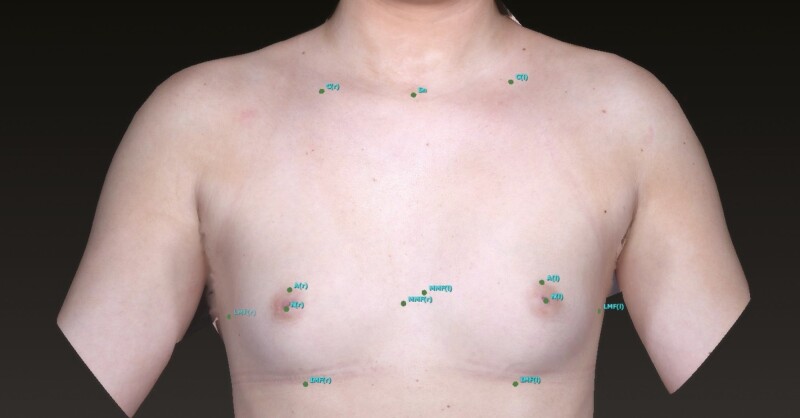
Pictured is a 39-year-old female. Vectra utilizes anatomic landmarks for developing the 3D image of a patient’s breasts. A, areola border; C, clavicle; IMF, inframammary fold; LMF, lateral mammary fold; MMF, medial mammary fold; N, nipple; Sn, sternal notch.

**Figure 2.**
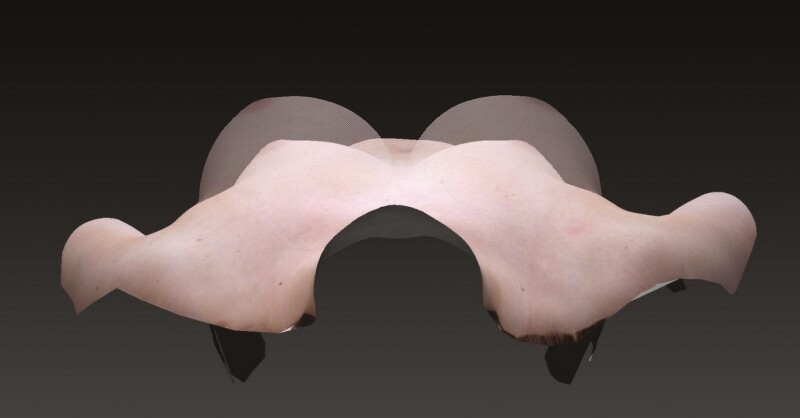
A 39-year-old female with a top-down view of her actual breast with proposed augmented breast image.

**Figure 3.**
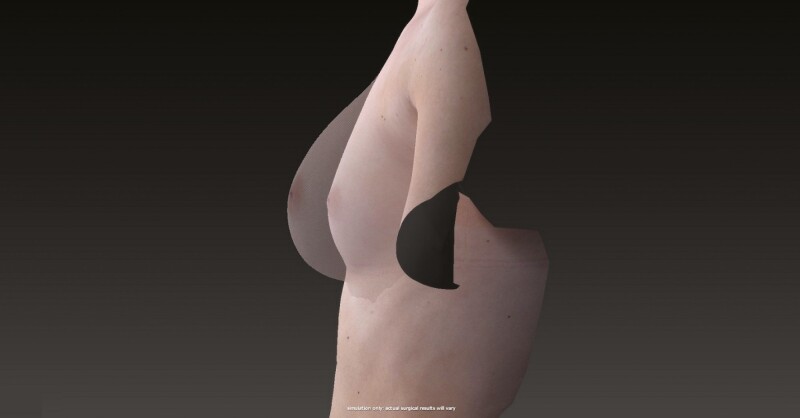
A 39-year-old female with a lateral view of her actual breast with proposed augmented breast image.

Our institution currently uses the VECTRA Volumetric 3D Surface Imaging System (Canfield Scientific Inc., Fairfield, NJ) to obtain preoperative 3D images for all breast augmentation patients. The VECTRA system uses 6 strategically placed cameras that take simultaneous photographs to generate a high definition, 3D color image of the patient. The photographs are taken in less than 2 ms, allowing for accurate and precise imaging despite subtle patient movement. The software then allows the patient’s likeness to be rotated for viewing from different angles and positions. The physician is able to demonstrate simulated breast augmentation outcomes with a variety of implant profiles and volumes. A ghost image is created to illustrate the difference between the projected outcome and the patient’s current breast size, shape, and volume (Figures 1-3).

### Data Collection

This study was reviewed by the Springfield Committee on Research Involving Humans Subjects (SCRHIS) and determined to be exempt from the standard approval process as it was considered an evaluation of the process of care. A retrospective review was conducted to identify patients at the Institute for Plastic Surgery at Southern Illinois University School of Medicine who underwent bilateral breast augmentation by a single surgeon N.Z.S. Those who presented for implant-based breast reconstruction were excluded from this study. Patients presenting over a 3.5-year period were included in the study. Those who presented after the purchase of the VECTRA 3D Surface Body Imaging System underwent preoperative imaging.

Patient-reported outcomes (PROMs) are an important component of breast surgery and evidence-based decision making. PROMs allow for the assessment of outcomes based on the quality-of-life factors that matter most to patients. Quality-of-life assessment and patient-perceived outcomes are especially important in the field of plastic surgery to determine treatment efficacy.^8,9^ Pusic et al established the BREAST-Q survey in 2007 as the only breast-specific outcome questionnaire with appropriate development and validation.^9^ The BREAST-Q was developed using international PROM measure guidelines and psychometric analysis.^8,9^ It focuses on 6 major topics to quantify patient satisfaction with breast surgery and health-related quality of life: satisfaction with breasts, satisfaction with the overall outcome, psychosocial well-being, sexual well-being, physical well-being, and satisfaction with care.^8^ Thus, we felt that the BREAST-Q Augmentation module would be the optimal tool for evaluating patient satisfaction in our study population.^8,9^

Identified patients were mailed a copy of the postoperative BREAST-Q breast augmentation module and an additional survey (Appendices A, B). The additional survey evaluated expected vs actual breast outcomes, confidence in implant size selection, communication with the surgeon, desire for 3D imaging, the effect of 3D imaging on surgeon selection, and decision to undergo surgery. Patients who did not respond to the initial mailing were sent a second copy of the surveys. Patient charts were reviewed to obtain patient age, time since surgery, history of previous breast augmentation, surgical complications, and implant type, size, and position.

Twelve patients were excluded from the study. Eight were excluded because they underwent unilateral implant exchange only. Two patients were excluded due to incomplete surveys, and 2 were excluded due to history of implant placement for breast reconstruction and congenital asymmetry.

### Statistical Analysis

In comparing the imaged and nonimaged groups, independent *t*-tests were used to analyze continuous variables (age, average implant size, time since surgery, and BREAST-Q scores), chi-squares tests of independence to analyze categorical variables (implant type, implant position, previous augmentation, and occurrence of any complication), and Wilcoxon rank-sum tests to analyze ordinal outcomes (size correlation score, shape correlation score, overall correlation score, confidence in implant size score, and communication with surgeon score). Results were considered statistically significant for *P* < 0.05.

Independent group’s *t*-tests, Wilcoxon rank-sum tests, and Pearson and Spearman correlation coefficients were used to identify predictors of the BREAST-Q and Southern Illinois University (SIU) survey scores. Differences in respondent and nonrespondent groups were compared using independent *t*-tests for continuous measures and chi-square tests of independence for categorical measures.

## RESULTS

A total of 120 patients were mailed surveys and 61 patients returned the surveys after either the first or second mailing for a response rate of 50.8%. Of the 61 respondents, 28 had preoperative imaging and 33 did not have preoperative imaging. After excluding several patients (see Data Collection), there were 26 patients in the imaged group (study group) and 28 patients in the nonimaged group (control group). There were no statistically significant differences between respondents and nonrespondents in terms of age, average implant size, time since surgery, implant type, implant position, history of previous augmentation, recorded complications, and history of 3D imaging.

### Demographics and Patient Characteristics of Study and Control Groups

All imaged participants were female with a mean age of 37.6 (range 24-69) while all non-imaged participants were female with a mean age of 46.0 (range 26-73). Statistically significant differences in age, time since surgery, and pocket placement were identified between imaged (study) and nonimaged (control) groups. The imaged group was found to be younger, have a shorter time since surgery, and more subpectoral implant placement. No statistically significant differences in implant size, implant type, history of previous breast augmentation, or history of postoperative complications were identified between the imaged and nonimaged groups. Complications included but were not limited to asymmetry, nipple contracture/herniation, capsular contracture, decreased nipple sensation, hypertrophic scarring, hematoma, and venous thrombosis (Table 1).

**Table 1.** Comparison of Patient Demographics Between Control and Study Groups

Demographic/characteristic	3D imaging	Mean score	*P*-value
Age	N	46.0 (range, 26-73)	0.022
	Y	37.6 (range, 24-69)	
Implant size (cc)	N	328 (range, 150-600)	0.74
	Y	322 (range, 210-560)	
Time since surgery (mo)	N	30.2 (range, 14-42)	0.0001
	Y	9.2 (range, 3-17)	
% Saline vs silicone implants	N	79	0.84
	Y	81	
% Subglandular vs submuscular pocket	N	44	0.0099
	Y	12	
% With previous breast augmentation	N	36	0.037
	Y	12	
% With postoperative complication(s)	N	60	0.081
	Y	35	

3D, three dimensional; N, no; Y, yes.

### Customized SIU Breast Augmentation Survey

While all reported values were higher for the imaged group, no statistically significant differences were found on the SIU survey (Table 2).

**Table 2.** Results of SIU 5-Point Scale Survey for Imaged and Nonimaged Groups

Customized SIU breast augmentation responses	3D imaging	Mean score	*P*-value
Size correlation score	N	3.69 (range, 1-5)	0.220
	Y	4.23 (range, 1-5)	
Shape correlation score	N	3.92 (range, 1-5)	0.359
	Y	4.31 (range, 1-5)	
Overall correlation score	N	3.58 (range, 1-5)	0.163
	Y	4.20 (range, 2-5)	
Confidence in implant size score	N	3.41 (range, 1-5)	0.297
	Y	3.88 (range, 2-5)	
Communication with surgeon score	N	4.22 (range, 1-5)	0.351
	Y	4.60 (range, 3-5)	

3D, three dimensional; N, no; Y, yes.

Several yes/no answer questions were also included. Approximately, 77% of study patients said given the option they would choose to have 3D breast imaging; 81% would recommend 3D imaging to a friend; 12% said that 3D imaging affected their choice of surgeon; and 27% said 3D imaging affected their decision to have surgery. Also, 65% reported imaging as helpful in both selecting implant size and predicting surgical outcome; 56% said imaging enhanced communication with the surgeon; and 74% of nonimaged patients said that they would want 3D imaging.

### BREAST-Q Augmentation Module Survey

The imaged group reported higher scores for satisfaction with outcome and surgeon as well as physical well-being on the BREAST-Q survey. The nonimaged group reported higher scores for satisfaction with breast, information, medical, and office staff as well as sexual and psychosocial & sexual well-being. However, none of these differences were found to be statistically significant (Table 3).

**Table 3.** Results of BREAST-Q Survey for Imaged and Nonimaged Groups

BREAST-Q augmentation responses	3D imaging	Mean score	*P*-value
Satisfaction with breast	N	69.21(range, 29-100)	0.997
	Y	69.19 (range, 20-100)	
Satisfaction with outcome	N	71.32 (range, 0-100)	0.947
	Y	71.85 (range, 20-100)	
Psychosocial well-being	N	83.14 (range, 38-100)	0.785
	Y	81.62 (range, 45-100)	
Sexual well-being	N	83.14 (range, 12-100)	0.541
	Y	81.62 (range, 42-100)	
Physical well-being	N	85.68 (range, 47-100)	0.489
	Y	88.15 (range, 72-100)	
Satisfaction with information	N	76.52 (range, 47-100)	0.606
	Y	73.31 (range, 14-100)	
Satisfaction with surgeon	N	87.22 (range, 38-100)	0.899
	Y	87.96 (range, 51-100)	
Satisfaction with medical staff	N	95.32 (range, 51-100)	0.804
	Y	94.42 (range, 55-100)	
Satisfaction with office staff	N	95.43 (range, 53-100)	0.268
	Y	91.08 (range, 58-100)	

3D, three dimensional; N, no; Y, yes.

### Effect of Demographics and Patient Characteristics on Survey Results

The use of silicone vs saline implants was associated with higher size correlation and psychosocial well-being scores. Submuscular vs subglandular pocket positioning was associated with a higher breast shape score. The presence of any complication was associated with lower sexual well-being scores. Increased age was associated with improved satisfaction with medical staff score. Increased time since surgery was associated with decreased breast outcome correlation, size correlation, and communication with surgeon scores.

## DISCUSSION

Three-dimensional imaging has many potential applications in the field of cosmetic and reconstructive breast surgery. Current 3D imaging systems employ laser scanning, stereophotogrammetry, or a combination of the two. Three-dimensional laser scanning uses objective measurements to determine postoperative changes in the augmented breast, which include breast position, volume, and shape.^10-15^ Three-dimensional imaging provides a unique method to assess and document breast surgery outcomes over time.^16^ Our VECTRA system was purchased at a cost of $38,000. Although 3D imaging technology offers many advantages, the cost of the system may be prohibitive for many plastic surgery practices.

Patient preference plays a vital role in implant selection. Various methods of implant selection have been described. Brody recommends having patients choose and fill a bra to a size that they are satisfied with. The padding is exchanged for a water-filled plastic bag to determine implant size.^17^ Tegtmeier developed an externally applied “mammary sizer” designed to be worn with different sized implants to give patients an indication of predicted postoperative size.^18^ Hidalgo and Spector recommend having patients wear a larger bra with their desired cup size and try different implants in the bra to choose an implant volume.^19^ De Runz et al found that 3D simulation is a useful aid for patients when envisioning their postoperative results, but that it should be combined with the traditional method proposed by Brody of trying on implants of different sizes in a bra.^20^ Other studies have demonstrated the accuracy of 3D simulation in predicted postoperative breast volume and surface contour to be >90% and 98.4% accurate, respectively.^21^ Donfrancesco et al found that a statistically significant number of patients felt that 3D simulation was very accurate in predicting aesthetic outcomes, aiding greatly in implant selection.^22^ Similarly, Overschmidt et al found that patients were more likely to seek out practices that offer 3D imaging when made aware of its existence. Yet, like the results of this study, 3D imaging was not found to affect outcomes in a statistically significant manner.^23^

Dealing with asymmetry is an essential component of breast surgery, as some degree of asymmetry is always present in the preoperative and postoperative breast patient. Liu et al used 3D scanning technology to identify and quantify breast asymmetry in 100 preoperative breast augmentation patients.^4^ All patients had some degree of asymmetry. Significant breast mound asymmetry was seen in 94% of the patients.^4^ Thus, 3D imaging is valuable to demonstrate existing asymmetry to patients before surgery and aids in surgical planning. The ability to demonstrate patient’s asymmetries preoperatively through 3D imaging was found to be of great benefit to the single surgeon’s breast augmentation practice in particular. While not captured by study data, several patients were able to visualize with VECTRA software that their desired result, most commonly in regard to breast shape, was not achievable with breast augmentation alone, whether due to existing asymmetries, significant ptosis, or preoperative unattractive breast shape. Many of these patients chose not to continue with breast augmentation or to undergo combined breast augmentation and mastopexy. In the authors’ opinion, counseling alone was not able to effectively communicate these differences in desired vs likely outcomes. Patients, who decided to pursue augmentation mastopexy after viewing their projected postoperative outcomes with augmentation alone, were not included in this study.

The results of both the BREAST-Q and SIU surveys demonstrated a trend toward improved patient satisfaction in several categories with the use of 3D imaging technology. BREAST-Q scores were improved, though not statistically significant, in regard to satisfaction with outcome and surgeon as well as physical well-being, and SIU survey scores were improved, though not statistically significant, for all measures. In both surveys, scores related to communication and satisfaction with surgeon were improved. Studies demonstrate a positive correlation between physician consultation time and patient satisfaction.^24^ Surgical consultation time is often increased when utilizing the VECTRA system and may have contributed to improved surgeon satisfaction and communication scores. The authors find that the ability to make the patient feel more involved in decision making through a visual process and increased time in consultation are some of the key advantages of 3D imaging, even if most of the other outcomes remain similar between imaged and nonimaged groups.

This study has several limitations. It is retrospective in nature and lacks randomization. Possible confounding variables include time since surgery and implant position, as these variables exhibited statistically significant differences between imaged and nonimaged groups (Table 1). The imaged group exhibited a statistically significant shorter time since surgery and greater subpectoral implant placement. Shorter time since surgery was associated with improved breast outcome correlation, size correlation, and communication with surgeon scores, suggesting that part of the trend toward improved scores in these categories in the imaged group may be attributed to shorter time since surgery. Similarly, greater subpectoral implant placement was associated with improved breast shape score, suggesting that part of the trend toward improved scores in this category in the imaged group may be attributed to greater subpectoral implant placement. Of note, the incidence of complications such as capsular contracture, rupture, and ptosis increase with increased time since surgery. The time since surgery was 30.2 months for the control group and 9.2 months for the study group. A study by Stevens et al found that 47% and 83% of capsular contractions occur within the first 2 and 4 years following breast augmentation, respectively.^25^ Finally, with power set at 0.80 for the SIU and BREAST-Q surveys, a sample size of approximately 300 and 2000 patients, respectively, would be required to detect differences in survey responses. Future studies would benefit from a larger sample size to determine a significant statistical significance, not just a trend.

## CONCLUSIONS

Three-dimensional imaging is a valuable tool in breast surgery for preoperative evaluation, patient communication, and patient satisfaction. Although our pilot study showed improvement in patient satisfaction and predicted outcome scores in the 3D imaged group, our results were not statistically significant. However, the use of 3D imaging often increases the amount of patient consultation time, which studies show translates to increased patient satisfaction with the surgeon. With the majority of patients reporting that they would choose 3D imaging and recommend it to a friend, 3D imaging appears to instill confidence in patients regarding both surgeon and implant selection.

## Supplementary Material

ojab005_suppl_Supplementary_Appendix_A

ojab005_suppl_Supplementary_Appendix_B
