# Supplementary material for: A Pilot Study Evaluation of 3-Dimensional Imaging in Cosmetic Breast Augmentation: Results of a Single Surgeon 3.5-Year Retrospective Study Using the BREAST-Q Questionnaire
Source: Aesthet Surg J Open Forum. 2021 Jan 25;3(1):ojab005. doi: 10.1093/asjof/ojab005 (PMC7953835; doi:10.1093/asjof/ojab005)
Supplement: ojab005_suppl_Supplementary_Appendix_A [file ojab005_suppl_supplementary_appendix_a.docx]

1. The following series of questions address how closely your breasts after your breast augmentation surgery match what you expected your breasts to be like. Please answer on a scale of 1 to 5, where 1 is completely different than what you expected and 5 is exactly what you expected.
   1. How closely does the **size** of your breasts after your breast augmentation surgery match the **size** you expected your breasts to be? Please circle one answer.

1 (totally different size than what I expected)

2

3

4

5 (exactly the size I expected)

- 1. How closely does the **shape** of your breasts after your breast augmentation surgery match the **shape** you expected your breasts to be? Please circle one answer.

1 (totally different shape than what I expected)

2

3

4

5 (exactly the shape I expected)

- 1. How closely does the **overall appearance** of your breasts after your breast augmentation surgery match the **overall appearance** you expected your breasts to be? Please circle one answer.

1 (totally different overall appearance than what I expected)

2

4

5 (exactly the overall appearance I expected)

1. Before you had your surgery, how confident were you that you picked the right implant size? Please answer on a scale of 1 to 5, where 1 is totally unsure and 5 completely sure. Please circle one answer.

1 (totally unsure)

2

3

4

5 (completely sure)

1. Did you have good communication with your surgeon regarding the details of your operation? Please answer on a scale of 1 to 5, where 1 is poor communication and 5 is perfect communication. Please circle one answer.

1 (poor communication)

2

3

4

5 (perfect communication)

1. Would you have wanted to have three-dimensional imaging of your breasts before your surgery? Please circle one answer.

Yes

No

I don’t know

1. Would the option of three-dimensional pictures of your breasts have influenced your decision to have your breast augmentation surgery? Please circle one answer.

Yes

No

I don’t know

1. Would the option of three-dimensional pictures of your breasts influence your choice of surgeon? Please circle one answer.

Yes

No

I don’t know

1. Please include any additional comments that you might have in the space provided below:
